# YidC and SecYEG form a heterotetrameric protein translocation channel

**DOI:** 10.1038/s41598-017-00109-8

**Published:** 2017-03-07

**Authors:** Ilie Sachelaru, Lukas Winter, Denis G. Knyazev, Mirjam Zimmermann, Andreas Vogt, Roland Kuttner, Nicole Ollinger, Christine Siligan, Peter Pohl, Hans-Georg Koch

**Affiliations:** 1 Institut für Biochemie und Molekularbiologie, ZBMZ, Faculty of Medicine, Albert-Ludwigs-Universität Freiburg, Stefan Meier Str. 17, Freiburg, 79104 Germany; 2 Fakultät für Biologie, Albert-Ludwigs-Universität Freiburg, Stefan Meier Str. 17, 79104 Freiburg, Germany; 3 Institute of Biophysics, Johannes Kepler University Linz, Gruberstrasse 40, A-4020 Linz, Austria; 4 Spemann-Graduate School of Biology and Medicine (SGBM), Albert-Ludwigs-Universität Freiburg, 79104 Freiburg, Germany

## Abstract

The heterotrimeric SecYEG complex cooperates with YidC to facilitate membrane protein insertion by an unknown mechanism. Here we show that YidC contacts the interior of the SecY channel resulting in a ligand-activated and voltage-dependent complex with distinct ion channel characteristics. The SecYEG pore diameter decreases from 8 Å to only 5 Å for the YidC-SecYEG pore, indicating a reduction in channel cross-section by YidC intercalation. In the presence of a substrate, YidC relocates to the rim of the pore as indicated by increased pore diameter and loss of YidC crosslinks to the channel interior. Changing the surface charge of the pore by incorporating YidC into the channel wall increases the anion selectivity, and the accompanying change in wall hydrophobicity is liable to alter the partition of helices from the pore into the membrane. This could explain how the exit of transmembrane domains from the SecY channel is facilitated by YidC.

## Introduction

**Figure 1:**
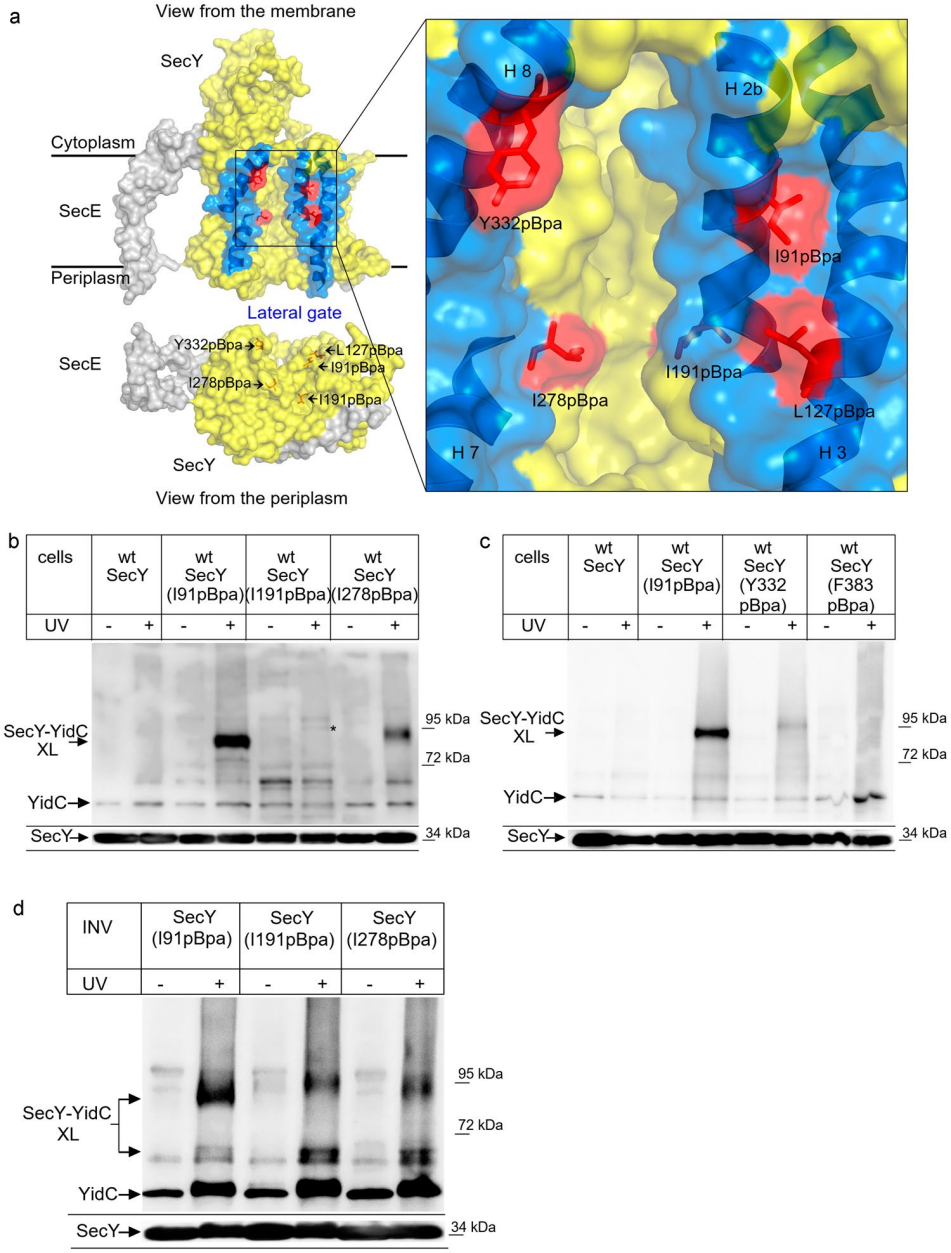
YidC contacts the channel interior of SecY. (**a**) Structure of the SecYE complex (left panel; PDB 3J01^63^) and the SecY channel interior and pore ring (right panel). ResiduesI91, L127, I191, I278 and Y332 where pBpa was inserted are indicated in red. The lateral gate is indicated in blue and the residues used for crosslinking are indicated. Residue F383 is at the back of the SecYEG complex and not visible in this front view. (**b**) *In vivo* crosslinking of Bl21 (wt) cells expressing SecY, either without pBpa insertion (SecY) or with pBpa inserted at the indicated positions. Crosslinking was induced by UV exposure of whole cells when indicated. SecY and SecY crosslinking products were purified after cell breakage and separated on SDS-PAGE. SecY-YidC crosslinking products were detected after western blotting using α-YidC antibodies. Indicated is the SecY-YidC cross-linking product (upper panel) and the SecY content in these cells, as revealed by α-SecY antibodies (lower panel). (*) indicates a weak UV-dependent SecY-YidC crosslinking product of SecY(I191pBpa). (**c**) *In vivo* crosslinking was performed and analyzed as in (**b**) with pBpa inserted at the indicated residues. (**c**) *In vitro* crosslinking with purified inner membrane vesicles (INV) (2.5 nM SecY) derived from BL21 cells expressing the indicated pBpa-containing SecY derivatives. Samples were processed as above. Crosslinking experiments were repeated at least three times and representative blots are shown.

The essential Sec translocon constitutes a universally^1, 2^ conserved protein-conducting channel which transports newly synthesized proteins across and into the bacterial cytoplasmic membrane or the eukaryotic endoplasmic reticulum membrane^1^. The Sec translocon consists of three core proteins, termed Sec61αβγ in eukaryotes or SecYEG in bacteria^1, 2^, which form the minimal membrane-embedded unit required for protein transport^3, 4^. SecY contains 10 transmembrane domains (TMs), which are arranged in two halves around a central channel^5^ (Fig. 1). A side view of SecY shows two vestibules that are separated by a central constriction called the pore ring. This constriction is further sealed by a short helix called the plug. A lateral opening is formed at the front of the SecYEG complex by TMs 2b, 3, 7 and 8 of SecY, through which signal sequences and substrate TMs are thought to exit the channel^6^. SecE appears to stabilize the two halves of SecY^7^ at the back of the SecYEG complex, while SecG seems to be specifically required during post-translational transport of secretory proteins into the periplasm^8^.

The Sec translocon is modularly organized^1^ and two additional membrane-embedded components, YidC and the SecDFYajC complex^9–12^, influence protein transport through the bacterial SecYEG complex. *E. coli* YidC is a conserved 60 kDa membrane protein insertase^13^ that can either function in concert with SecYEG^14–16^ or autonomously during the insertion of membrane proteins^17, 18^. During SecYEG-dependent insertion, YidC is suggested to facilitate the lateral partitioning and folding of TMs exiting SecY^12, 19^. YidC has been shown to interact in a substrate-dependent manner with the surface of the lateral gate of SecY^20^. However, the mechanism by which YidC aids the release of TMs from the SecY channel is unknown.

The auxiliary SecDFYajC complex was also shown to interact with the SecYEG complex, but its exact function is unknown^9^. SecD is a 66 kDa membrane protein that consists of six TMs and a large periplasmic loop. The topology of the 36 kDa SecF is quite similar to SecD, but the periplasmic loop size is smaller^21^. Both SecD and SecF belong to the resistance-nodulation-cell division (RND) superfamily of membrane proteins, often associated with proton motive force (pmf)-driven transport processes^22^. The TMs in a recently solved crystal structure of the SecDF fusion protein from *Thermus thermophilus* show a similar arrangement as in AcrB, a proton/multi-drug antiporter of the RND family^22^. SecDF was proposed to function as a pmf-powered chaperone that supports ATP-independent steps during protein transport^22^. YajC is a small, single-spanning membrane protein encoded upstream of SecD in the *yajC-secDF* operon. Its expression level is tenfold higher than that of SecDF^23^, which might explain the presence of YajC complexes that do not contain SecDF^9, 24^. SecDFYajC was cross-linked to SecG in a complex consisting of SecYEG, YidC and SecDFYajC^25^, supporting an earlier observation of a functional SecDFYajC-SecG interaction^26^.

SecDFYajC is possibly involved in tethering YidC to SecYEG. This was deduced from the observation that co-purification of YidC with SecYEG was detected upon SecDFYajC overexpression^10^ and that SecDF forms a complex with YidC^27^. Nonetheless, the interaction between YidC and the lateral gate of SecY is not significantly influenced by the absence of SecDF^20^.

In the current study, we uncovered that YidC not only interacts with the surface of SecY’s lateral gate, but that it also contacts the channel interior of SecY. Electrophysiology analyses revealed that YidC directly influences the channel properties of the SecYEG channel by altering the channel cross-section and anion selectivity. Our data demonstrate that upon complexation with SecYEG, YidC becomes part of the protein-conducting channel.

## Results

### YidC is in contact with the SecY channel interior

YidC’s position in front of the lateral gate of SecY^20^ likely explains the observation that during insertion, membrane proteins were found at the SecY-YidC interface^12, 19^. Crosslinking data indicate that the SecY-YidC contact is influenced by ribosome-nascent chains (RNCs), demonstrating that the SecY-YidC interaction is different in resting and active translocons^20^. For analyzing these conformational changes, we employed *in vivo* site-directed photo-crosslinking using the phenylalanine derivative para-benzoyl-L-phenylalanine (pBpa)^28^. pBpa was incorporated into residues I91 (TM2b), L127 (TM3), I191 (TM5), I278 (TM7), Y332 (TM8) and F383 (TM9) (Fig. 1a). I91 and L127 are located on the surface of the lateral gate and have been shown to contact YidC^20^. Residues I191 and I278 are part of the internal pore ring and thus located inside of the protein conducting channel (Fig. 1a). Y332 is also part of the lateral gate, but located on the other site compared to I91 and L127. Finally, residue F383 is located at the back of SecY and thus not in vicinity to the lateral gate.

UV-exposure of *E. coli* cells expressing pBpa-containing SecY derivatives induces the formation of a covalent bond between SecY and any protein in close proximity. We observed a strong crosslink between residue 91 and YidC at approx. 90 kDa (Fig. 1b), which was recognized by α-YidC antibodies and not present when cells expressing SecY without pBpa were UV-exposed. This crosslinking product had been observed before^20^ and was identified by mass spectrometry as a SecY-YidC crosslinking product^20^. This confirms YidC’s position close to the lateral gate. We also observed a crosslink between residue 278 and YidC, suggesting that YidC is not only in contact with the surface of the lateral gate, but that it actually extends into the SecY channel (Fig. 1b). We did not observe a strong crosslink to YidC from residue 191 *in vivo*, although we did detect a very weak UV-dependent band that was recognized by YidC antibodies (Fig. 1b, *). This band migrated slightly higher than the SecY-YidC crosslink products from positions 91 and 278. Irregular migration of SecY crosslink products on SDS-PAGE is commonly observed and probably reflect different folding states of the crosslink product^20, 29–31^. For position 332 of the lateral gate, we observed only a very weak crosslink product and position 383 at the back of SecY did not show any crosslink to YidC (Fig. 1c). Thus, our data confirm the contact between the lateral gate of SecY and YidC, but surprisingly also show that YidC is in contact with interior of the SecY channel. No crosslink was observed between the back of SecY and YidC, indicating that YidC is primarily in contact with the front of the SecYEG complex.

*In vivo* crosslinking is advantageous for monitoring protein-protein interactions in living cells, but since it is unsynchronized, only rather strong interactions are detected. We therefore also analyzed the interaction between YidC and the channel interior by *in vitro* crosslinking. Inner membrane vesicles (INV) of cells expressing pBpa-containing SecY derivatives were purified and subsequently UV exposed for activating pBpa. This approach confirmed the 90 kDa SecY-YidC crosslink product for residue 91 but also showed an additional weak crosslink product at approx. 70 kDa (Fig. 1d). This band was previously identified by mass spectrometry as additional SecY-YidC crosslink product^20^. The occurrence of two crosslink products either indicates that this SecY residue is in contact with two different regions of YidC. Such a position-dependent mobility of pBpa crosslink products on SDS-PAGE is frequently observed for SecY and is assumed to reflect different three-dimensional structures of the crosslink products^29, 31^. Alternatively, the lower band could reflect a proteolytic cleavage product. In particular SecY is subject to proteolysis under *in vitro* conditions^32, 33^, which would explain why the 70 kDa band is primarily observed under *in vitro* conditions.

*In vitro*, the 90 kDa and 70 kDa crosslink products were also observed for residues 278 and 191, although the latter showed only very weak crosslinks to YidC *in vivo*. As *in vivo*, the *in vitro* 90 kDa YidC crosslink product from residue 191 displayed slightly reduced mobility on SDS-PAGE.

**Figure 2:**
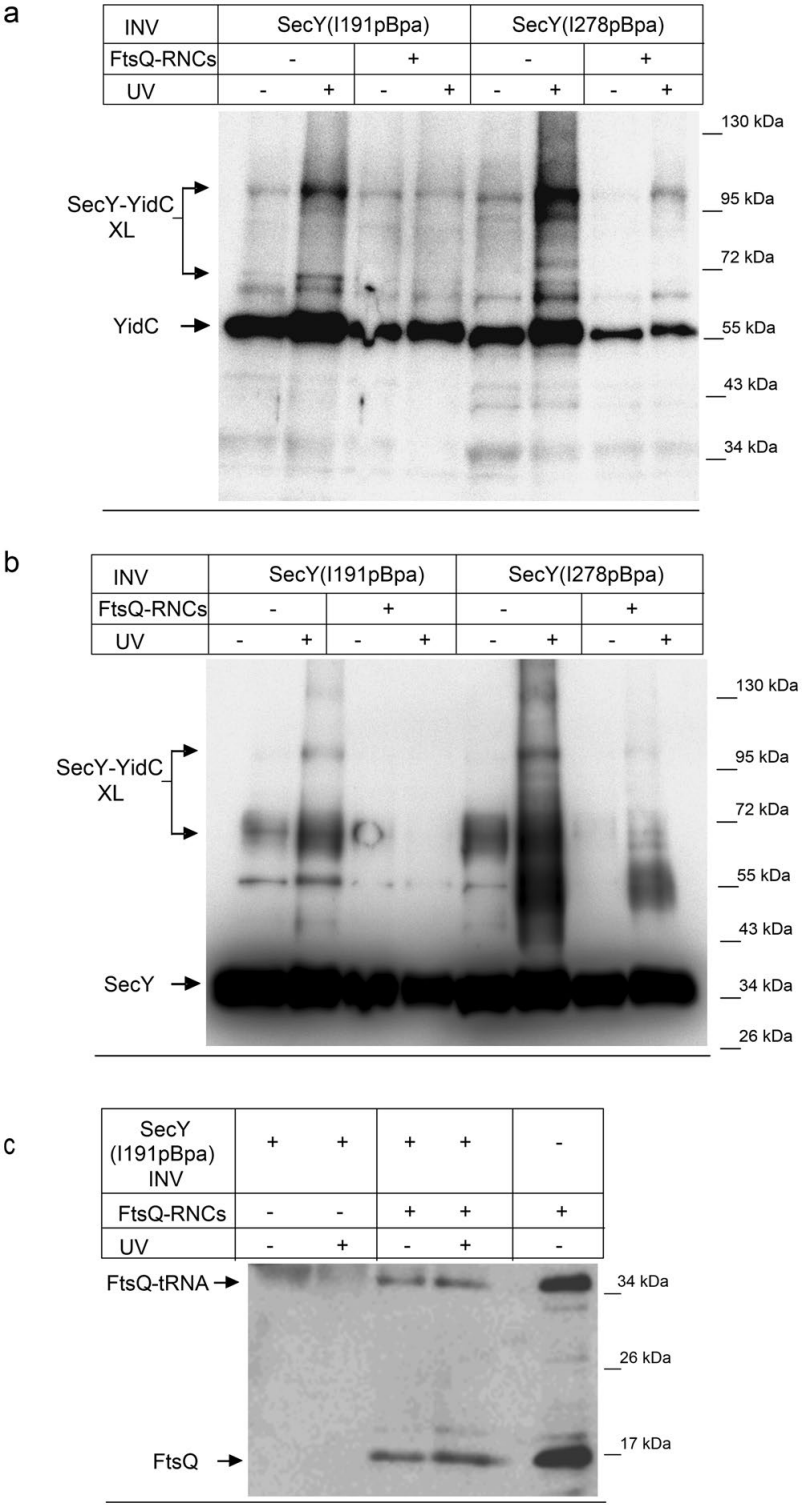
YidC loses contact to the SecY channel interior in the presence of a nascent membrane protein. (**a**) *In vitro* crosslinking using SecY(I191pBpa) and SecY(278pBpa) INV (2.5 nM SecY) was performed in the absence or presence of FtsQ-RNCs (2.5 nM) as indicated. Samples were processed as described in Fig. 1 and decorated with antibodies against YidC. The 90 kDa SecY-YidC crosslink product is indicated. (**b**) The samples described in (**a**) were decorated with antibodies against SecY after western blotting and the 90 kDa SecY-YidC crosslink product is indicated. (**c**) Western blot of the material shown in (**a**) for SecY(I191pBpa) INV using antibodies against the HA-tag present at the N-terminus of the FtsQ-RNCs. The upper band corresponds to FtsQ that is still tRNA-bound (FtsQ-tRNA) and the lower band to FtsQ that was released during sample preparation for SDS-PAGE (FtsQ). Crosslinking experiments were repeated at least three times and representative blots are shown.

The different crosslink pattern of SecY(I191pBpa) *in vivo* and *in vitro* could reflect the fact that in sucrose-gradient purified INV most of the SecYEG channels are in a closed or inactive state, while the *in vivo* crosslinking monitors crosslinking of both active and inactive SecYEG channels. Thus, the observation that SecY(I191pBpa) forms strong crosslinks to YidC only *in vitro* could indicate that access of YidC to the channel interior is restricted in the presence of a nascent chain. This was further analyzed by performing the *in vitro* crosslink experiment in the presence of purified FtsQ-RNCs. FtsQ is a single spanning membrane protein that is inserted into the *E. coli* membrane via the SecYEG-YidC pathway^10, 34^. Upon UV exposure of INV containing either SecY(I191pBpa) or SecY(I278pBpa), the two crosslink products at 90 kDa and 70 kDa were detected by α-YidC antibodies (Fig. 2a) and also by α-SecY antibodies (Fig. 2b), although the specificity of the α-SecY antibodies was lower. When these INV were pre-incubated with FtsQ-RNCs before UV-exposure, the crosslink products became significantly weaker for I278 and were almost absent for I191 (Fig. 2). In this experimental setting, we observed weak UV-independent crosslink formation, which is probably the result of light exposure during sample preparation and incubation. The presence of FtsQ-RNCs in the assay was monitored for SecY(I191pBpa)-INV by western blot using antibodies against the N-terminal HA-tag of the FtsQ-RNCs. This confirmed the presence of both FtsQ and the FtsQ-tRNA species in the RNC-containing sample (Fig. 2c).

Although all samples contained comparable SecY amounts as verified by western blotting (see Figs 1 and 2), we noticed that in all UV-exposed samples slightly more YidC co-purified with SecY, compared to the non-exposed sample. Co-purification of YidC with SecY has been observed before^10, 20^ and supports the conclusion that both proteins interact in the *E. coli* membrane. The increased co-purification of YidC with SecY after UV exposure could reflect the observation that YidC exists as both monomer and dimer^15, 27, 35^ in the *E. coli* membrane. Therefore a crosslink of SecY to one copy of the YidC dimer will likely increase the amount of YidC that is found co-purifying with SecY. When samples were pre-treated with 50 mM DTT, the amount of YidC co-purifying with SecY was comparable between the UV-treated and non-treated sample (Supplementary Fig. S1). The reasons for this obviously redox-sensitive interaction between SecY and YidC upon UV-exposure were not further analyzed in the current study.

The observed interaction of YidC with the channel interior of SecY could reflect a YidC molecule in the process of being co-translationally inserted via SecY. The size of the crosslink product, however, suggests that SecY is crosslinked to full length YidC, which would not be expected if YidC-RNCs were in contact with SecY. These highly purified INV are furthermore almost free of RNCs^36^. In summary, these *in vivo* and *in vitro* data indicate that YidC not only contacts SecY at the surface of the lateral gate, but that it is also in contact with the channel interior until it is reoriented by the presence of a nascent membrane protein.

### Channel properties of the YidC-SecYEG channel

**Figure 3:**
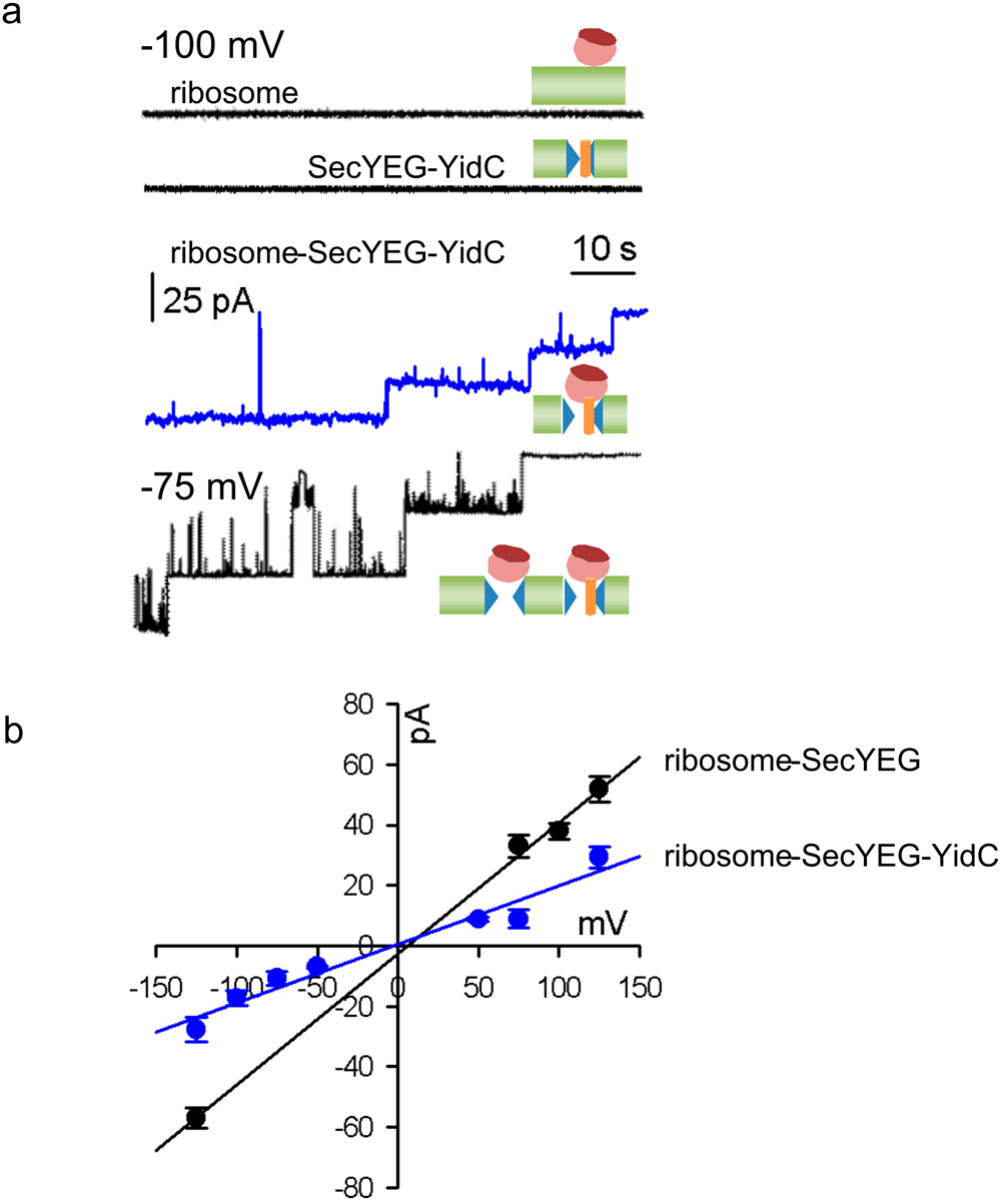
Single channel conductance of the ribosome-SecYEG-YidC complex. (**a**) Conductance measurements in the presence of (i) both bare liposomes and ribosomes (150 nM, upper black trace) or (ii) SecYEG-YidC proteoliposomes (2.5 nM SecYEG) in the absence of ribosomes (lower black trace) showed electrically silent planar bilayers. SecYEG and YidC were present at 1:1 molar ratio and the final protein concentration was 5 μM. Ribosome binding to the YidC-SecYEG complex induced single channel activity. Representative current traces show the subsequent closure of three single channels (blue trace) at −100 mV. Co-reconstitution of YidC with a 3-fold excess of SecYEG yielded a mixture of larger SecYEG and smaller SecYEG-YidC channels (lower black trace). Measurements were obtained from free-standing bilayers formed from *E. coli* polar lipid extract at symmetrical salt concentrations of 150 mM KCl. (**b**) Dependence of the single channel current on the transmembrane voltage of the ribosome-SecYEG (black) and the ribosome-YidC-SecYEG complex (blue). The slope of the linear regression gives single channel conductances for SecYEG and YidC-SecYEG of 439 pS and 195 pS, respectively. Ribosomes were present at a concentration of 150 nM.

Since a protrusion of YidC into the SecY channel is likely to decrease the channel lumen, we measured the single channel conductance *g* of SecYEG- or SecYEG-YidC complexes. To perform the electrophysiology measurements we first co-reconstituted SecYEG and YidC into proteoliposomes and then formed protein-containing monolayers on top of the proteoliposome suspensions. Two of these monolayers were then folded into a planar bilayer within the aperture of a Teflon septum^37^. This method of protein reconstitution into planar bilayers does not require the SecYEG channels in the proteoliposomes to be in their active state – in contrast to the widely used assays of proteoliposome fusion to planar lipid bilayers^38^. It thus allowed us to show that the YidC-SecYEG complexes, reconstituted in a 1:1 ratio, were closed in their resting state (Fig. 3a, 2^nd^ black trace). We recently discovered that ribosomes are able to open the SecYEG channel at low membrane potentials^39^. Thus, the SecYEG channel is both ligand dependent and voltage activated. At physiological membrane voltages, *i.e.* at about −130 mV, the channel is closed and ligands are unable to open it^40^. Most of the ribosome-bound SecYEG channels open upon membrane depolarization, *i.e.* at half the physiological value of the membrane potential. Channel opening is reversible, *i.e.* the channels close again upon restoration of more negative potentials. Opening of the SecYEG channel by ribosomes was not observed with a SecYEG mutant complex, in which two conserved arginine residues within the cytosolic loop C4 were replaced by glutamate (Supplementary Figs 2 and 3). This demonstrates that channel opening by ribosomes requires a specific SecY-ribosome interaction. The addition of ribosomes to just lipid bilayers did also not increase current, demonstrating that ribosomes are free of any pore-forming contaminant (Fig. 3a, 1^st^ black trace). In line with the recently discovered ability of ribosomes to open the SecYEG channel at low membrane potentials^39^, channel activity exclusively appeared upon ribosome addition. Figure 3a shows how three such ribosome-bound YidC-SecYEG channels close one after the other (blue trace). Co-reconstitution of YidC with a 3-fold excess of SecYEG yielded a mixture of larger SecYEG- and smaller SecYEG-YidC channels (Fig. 3; lower black trace). We obtained the single channel conductivity g by plotting the single channel current as a function of the applied voltage (Fig. 3b and Table 1). The *g*-value of ribosome-activated SecYEG channels was 1.6 ± 0.1 pS/mM, which is in perfect agreement with previously obtained results^39^. However, for the SecYEG-YidC complex we determined a significantly lower *g*-value of 0.9 ± 0.2 pS/mM, which is in line with a protrusion of YidC into the SecYEG lumen and verifies the crosslinking data between YidC and SecY-residue 191.

**Table 1:** Translocation channel diameter *d* and selectivity *r* of SecYEG and SecYEG-YidC complexes.

	ψ_r_ (mV)	*r*	$\tilde{g}$ (pS/mM)	*d* (Å)
SecYEG + ribosomes	−7.2 ± 2.3	2 ± 0.5	1.6 ± 0.1	8.4 ± 0.5
SecYEG + RNC_FtsQ_	−6.7 ± 1.1	1.9 ± 0.2	1.7 ± 0.1	8.6 ± 0.5
SecYEG/YidC + ribosomes	−9.2	2.5 ± 0.4	0.9 ± 0.2	5.1 ± 0.6
SecYEG/YidC + RNC_FtsQ_	−15 ± 3.9	5.3 ± 3.15	1.75 ± 0.1	8.1 ± 0.5

The reversal potential *ψ*_r_ was obtained by plotting the single channel current as a function of membrane voltage (Fig. 3b). Anion to cation permeability ratios *r* (mean ± standard deviation) were calculated according to Eq. 1. $\tilde{g}$ is the single-channel equivalent conductance, which is defined as the ratio of the single channel conductance *g* and the average ionic strength of solution in both the *cis* and *trans* compartments and *d* is the pore diameter calculated according to Eq. 2.

**Figure 4:**
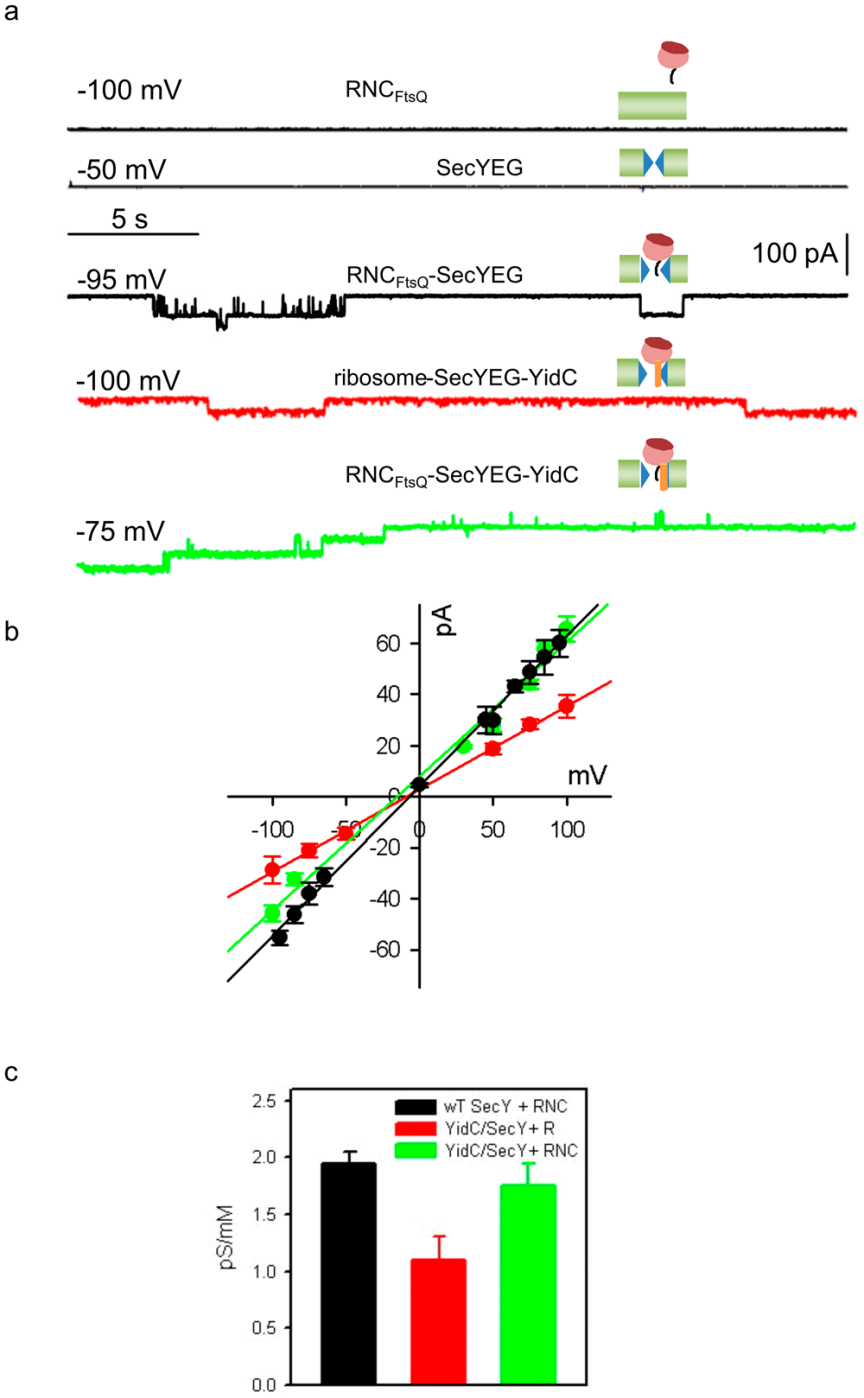
Single channel conductivity of the RNC_FtsQ_-SecYEG-YidC complex. Planar bilayers were formed from *E. coli* lipid. Fusion of proteoliposomes (5 μM final protein concentration, molar ratio SecYEG-YidC 1:1) to the membrane was facilitated by a transmembrane salt gradient of 435:150 mM KCl. (**a**) Channel activity was measured in the presence of liposomes and FtsQ-RNCs (5 nM, upper black trace), in the presence of SecYEG proteoliposomes (2.5 nM SecYEG; 2^nd^ black trace). Binding of RNC_FtsQ_ (5 nM) to either SecYEG proteoliposomes (3^rd^ black trace) or SecYEG-YidC proteoliposomes (green trace) opened the reconstituted translocons for ions. Channel opening by non-translating ribosomes (150 nM) was also analysed for SecYEG-YidC complexes (red trace). Representative current traces were recorded at the indicated transmembrane potential (**b**) The corresponding current-voltage characteristics are shown for the RNC_FtsQ_-SecYEG (black), the RNC_FtsQ_-YidC-SecYEG (green), and the ribosome-YidC-SecYEG complexes (red). Deriving the corresponding single channel conductances from the slopes of the linear regressions yielded 587, 525, and 324 pS, respectively. The respective ψr values amounted to 6.7, −15, and −9.2 mV. (**c**) The single channel equivalent conductances of the three complexes are shown in the same color-code.

Crosslinking between SecY’s residue 191 and YidC was only observed under *in vitro* conditions when the YidC-SecYEG complex was in its resting state. When actively engaged in protein transport, *i.e.* bound to translating RNCs, crosslinking to 191 was not observed, suggesting that YidC was expelled to the rim of the complex. This movement of YidC in the presence of RNCs should also be reflected by an increased *g*-value – provided the pore is not blocked by the nascent chain. In contrast to the experiments in Fig. 3, we applied a salt gradient across the membrane to test for ion selectivity of the complex, which would manifest itself in a reversal potential, *ψ*_r_. As a side effect, the salt gradient drives the fusion of proteoliposomes with planar bilayers prompting us to use these fusion events for reconstituting the SecYEG or the YidC-SecYEG complexes into the planar bilayers. Since fusion only occurs when the channels in the proteoliposomes conduct the osmolyte, we did not observe any channel activity in the absence of FtsQ-RNC (Fig. 4a, 2^nd^ black line). We did also not observe channel activity by just adding FtsQ-RNCs to bare bilayers (Fig. 4a, 1^st^ black line). In the presence of FtsQ-RNC, proteoliposome fusion served to insert open channels into the planar bilayer. Binding of FtsQ-RNCs or high concentrations of non-translating ribosomes opened the reconstituted SecYEG channels (Fig. 4a, black and red traces) and FtsQ-RNCs opened also the SecYEG-YidC channels (Fig. 4a, green trace). Under the conditions of the experiment, we did not observe channels that could be attributed to YidC alone. However, in complex with ribosomes or RNCs, YidC shows channel activity. These channels are distinct from both SecYEG and SecYEG-YidC channels, *e.g.* their single channel conductivity amounts to only 1.3 pS/mM when measured under the same conditions as the traces from Fig. 4 (to be reported elsewhere).

We obtained *g* of the FtsQ-RNC-YidC-SecYEG-complex by recording the single channel current at different membrane potentials (Fig. 4b). The addition of FtsQ-RNCs to the YidC-SecYEG complex resulted in an increased *g*-value of 1.75 ± 0.1 pS/mM, which was identical to the *g*-value of the FtsQ-RNC activated SecYEG complex (Fig. 4b). This further supports our hypothesis of a substrate-induced relocation of YidC from the channel interior to the rim. One possible explanation for identical *g* values is that YidC is no longer part of the channel wall and only contacts SecY residues at the outer surface of the channel. However, this is at odds with the observed change in the anion (Cl^−^) to cation (K^+^) permeability ratio *r* (P_Cl_/P_K_). For the RNC_FtsQ_-YidC-SecYEG complex, we measured ψ_r_ values of −15 ± 3.9 mV, while the FtsQ-RNC-SecYEG complex showed values of *ψ*_r_ −6.7 ± 1.1 mV. The corresponding *r* values of 5.1 ± 3.15 for the FtsQ-RNC-YidC-SecYEG complex and 1.9 ± 0.2 for the FtsQ-RNC-SecYEG complex indicate that the pore-lining residues are different in the two complexes. It appears likely that a positively charged YidC residue lines the pore thereby augmenting ion selectivity.

### SecDF depletion and the proton motive force stabilize the interaction of YidC with the channel interior of SecY

**Figure 5:**
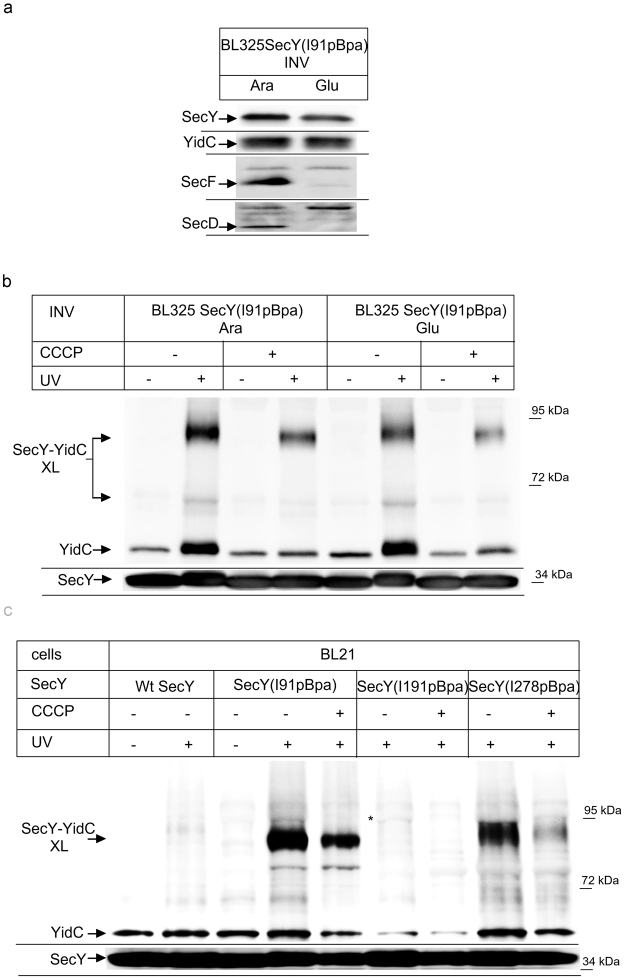
YidC’s interaction with SecY’s lateral gate is independent of SecDFYajC, but the interaction with both the lateral gate and the channel interior requires the pmf. (**a**) SecY(I91pBpa) was expressed in the conditional SecDFYajC-depletion strain BL325 and cells were grown either in the presence of arabinose for SecDFYajC induction or in the presence of glucose for SecDFYajC depletion. INV were isolated and the steady-state amounts of SecY, YidC, SecD and SecF were monitored by immune detection using the appropriate antibodies. (**b**) The INV as in (**a**) were used for *in vitro* crosslinking. When indicated, the protonophore CCCP was added at a final concentration of 100 μM. (**c**) *In vivo* crosslinking of Bl21 cells expressing either SecY without pBpa (wt) or SecY derivatives where pBpa was inserted at the indicated positions. When indicated, CCCP (100 μM final concentration) was added before UV-induced crosslinking. Experiments were repeated at least three times and representative blots are shown.

The interaction between SecY and YidC was reported to be dependent on the SecDFYajC complex^27, 41^, but SecDFYajC depletion had only a minor effect on the crosslink between YidC and the lateral gate of SecY^20^. To analyze whether SecDF influences YidC’s contact to the channel interior, we employed the conditional SecDF depletion strain *E. coli* BL325, which carries *secDF* under arabinose promoter control^9^. We prepared INV from *E. coli* BL325 cells expressing SecY(I91pBpa), grown either in the presence of arabinose or in the presence of glucose. In INV from glucose grown cells, SecD and SecF were undetectable by western blotting using α-SecD or α-SecF antibodies (Fig. 5a), while the cellular levels of SecY or YidC did not significantly change (Fig. 5a). When these INV were UV-exposed, a 90 kDa YidC crosslink product was detectable in both types of INV (Fig. 5b). In SecDF-depleted cells, the crosslink product was only slightly weaker, which confirms the previous conclusion that the SecY-YidC interaction persists even in the absence of SecDF^20^.

SecDF was suggested to be involved in pmf-dependent steps during protein transport. We thus also analyzed the effect of the protonophore CCCP (Carbonylcyanid-m-chlorophenylhydrazon) which collapses the proton gradient across the cytoplasmic membrane. The addition of CCCP reduced the amount of the SecY-YidC crosslinking product both in SecDF-containing and in SecDF-depleted cells (Fig. 5b). Thus, although the pmf appears to stabilize YidC’s interaction with SecY’s lateral gate, the effect is not drastically different in the presence or in the absence of SecDF.

The stabilizing effect of the pmf on the SecY-YidC crosslink was also observed in *E. coli* BL21 cells *in vivo*. The SecY-YidC crosslink products were significantly weaker in the presence of CCCP for residues 91 and 278 (Fig. 5c). For residue 191, we only found a weak *in vivo* SecY-YidC crosslink product, but this crosslink also seemed to be CCCP sensitive (Fig. 5c).

**Figure 6:**
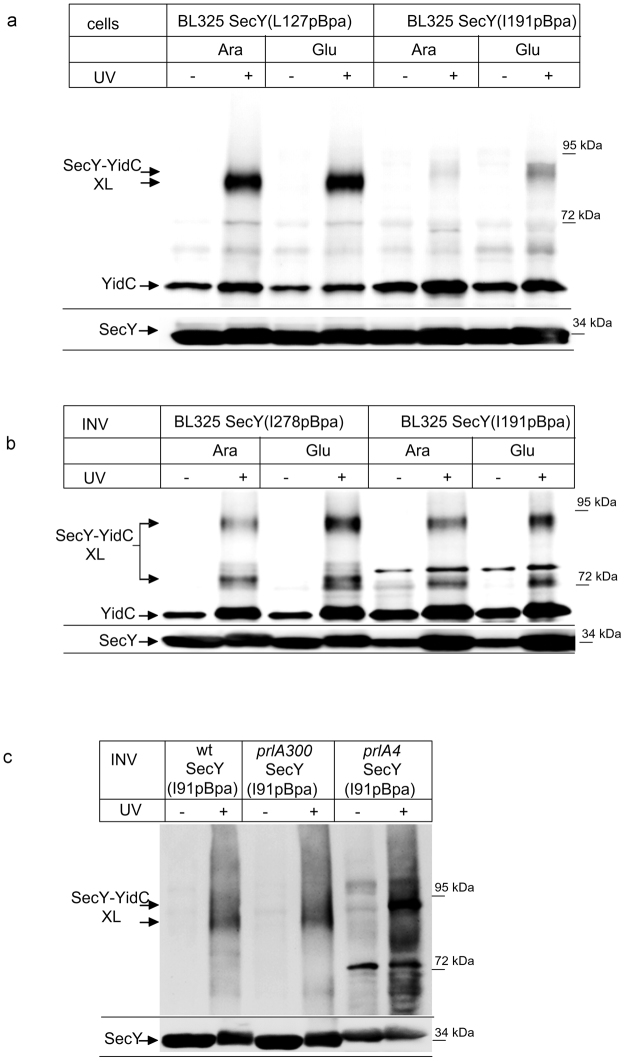
The depletion of SecDF enhances the interaction of YidC with the SecY channel interior. (**a**) *In vivo* crosslinking of SecDF-containing and –depleted BL325 cells expressing SecY derivatives with pBpa insertion at the indicated conditions. (**b**) *In vitro* crosslinking with INV derived from the indicated cells. (**c**) Crosslinking was performed with INV of *E. coli* BL21 cells expressing a plasmid-encoded wild type SecY (wt) or the plasmid encoded *prlA300* or the *prlA4* SecY derivatives, carrying each pBpa at position I91 in TM2b of SecY. Indicated is the SecY-YidC cross-linking product (upper panel) and the SecY content in these INV, as revealed by α-SecY antibodies (lower panel). Experiments were repeated at least three times and representative blots are shown.

SecDF depletion had no significant effect on the interaction of YidC with the surface of SecY’s lateral gate (position 91 of helix 2B)^20^ and this was also observed for position 127 within helix 3 of the lateral gate (Fig. 6a). It should be noted that we had previously observed two additional crosslink products for position 127 *in vivo*, which were by mass spectrometry also identified as SecY-YidC crosslinking products^20^. These products were not visible here, which is probably related to differences between *E. coli* BL21(C43) used previously and strain BL325 used here. We then analyzed whether SecDF influenced the interaction of YidC with the channel interior by performing *in vivo* crosslinking from residue 191, deep inside the Sec channel. As shown before for wild type cells (*c.f.* Fig. 5c), the insertion of pBpa into this residue only produced a very weak *in vivo* crosslinking product in SecDF-containing BL325 cells (Fig. 6a). In the absence of SecDF, however, a significant SecY(191pBpa)-YidC crosslink product was observed (Fig. 6a).

Whether the depletion of SecDF indeed promotes the interaction of YidC with the SecY channel interior was further confirmed by *in vitro* crosslinking. We isolated INVs of BL325 cells expressing either SecY(191pBpa) or SecY(278pBpa) from arabinose- or glucose-containing cultures and UV exposed them. For both position 278 and I191, we observed a significant increase of the SecY-YidC crosslink product in the absence of SecDF (Fig. 6b). Thus, both *in vivo* and *in vitro*, YidC has preferred access to the channel interior, in the absence of SecDFYajC.

In contrast to previous reports^25, 27, 41^, our data indicate that SecDF is not required for the SecY-YidC interaction and that YidC even has preferred access to the channel interior when SecDF is missing. This discrepancy is probably explained by the fact that we used a conditional SecDF depletion strain, while SecDF-overexpressing strains were often analyzed for a possible role of SecDF on SecY-YidC interaction^10, 25^. Intriguingly, the overexpression of SecDF induces a *prlA4* phenotype^11, 23^, which is connected with a weakened association between the subunits of the SecYEG translocon^42, 43^ and a SecYEG mediated ion leak^44^. We therefore analyzed whether the SecY-YidC interaction was influenced by the *prlA4* allele of SecY. pBpa insertion at position 91 of wild type SecY confirmed the 90 kDa SecY-YidC crosslinking product in INV (Fig. 6c). The same crosslinking product was observed when pBpa insertion at position 91 was combined with the *prlA300* allele of SecY, which carries a mutation within the plug domain^45^. However, combining the pBpa insertion at position 91 with the *prlA4* allele significantly increased SecY-YidC crosslinking (Fig. 6c). This was even more significant, because the expression level of SecY(prlA4-I91pBpa) was significantly lower than the SecY(I91pBpa) or SecY(prlA300-I91pBpa) expression levels (Fig. 6c, lower panel). The *prlA4* allele also displayed a slightly reduced electrophoretic mobility, which explains why the *prlA4* SecY-YidC crosslink product migrated slightly higher. These data demonstrate that the *prlA4* mutation of SecY stabilizes YidC’s interaction with SecY’s lateral gate. This probably explains why the SecY-YidC interaction is stronger^10, 27^ in SecDF overexpressing cells that display a *prlA4* phenotype^11^.

## Discussion

The association of YidC with the SecYEG translocon in *E. coli* was first shown by co-purification^10^ and was confirmed by *in vivo* crosslinking, which demonstrated that YidC crosslinked to the lateral gate of SecY^20^. The initial model of a sequential hand-over of a TM from SecY to YidC^12, 19^ assumed that YidC binds to an emerging TM outside of the SecY channel. However, we found that YidC is essentially part of the channel and thus the protein conducting channel is not only formed by SecY but rather by a SecY-YidC complex. Protrusion of YidC into the pore is demonstrated i) by crosslinks between YidC and SecY’s pore ring residues, ii) by the reduced single channel conductance of the translocation pore in YidC’s presence, and iii) by a YidC-induced increase in anion selectivity.

According to our conductivity measurements, the SecY pore ring forms an opening of approximately 8.4 ± 0.5 Å in the ribosome bound state (Table 1). Upon accommodation of a nascent chain, the pore ring assembly is partially disrupted by movement of TM10^46^, but ensemble measurements show no major plug movement^46, 47^. In contrast, single molecule measurements reveal conductivity, indicating that the plug moves out of the lumen in a fraction of channels and/or for a fraction of time^40^. Whatever the status of the plug, movements of TM2, TM7 and TM10 generate a continuous conduit including the pore and the lateral gate^46–48^. This allows substrate TMs to exit the channel, and probably also allows YidC to enter it. *In vivo* crosslinking is unsynchronized and covers YidC’s interaction with SecYEG channels that harbour a nascent chain and with empty SecYEG channels. This is different *in vitro*, where only a minority of SecYEG channels is likely to contain a nascent chain. The YidC crosslink product to residue 191 deep inside the channel was clearly detected, but only under *in vitro* conditions and this result suggests that YidC moves out of the channel interior in the presence of a nascent chain. This was demonstrated by the loss of the crosslink product in the presence of a nascent membrane protein and by conductivity measurements that revealed an increase in the YidC-SecYEG channel lumen from 6.2 ± 0.6 to 8.1 ± 0.5 Å in the presence of a stalled FtsQ chain. In the proteoliposomes used for the electrophysiology measurements, the orientations of SecYEG and YidC are difficult to control and they likely contain the proteins also in a mixed orientation. However, as the ribosome binding site of SecY is on the cytosolic phase of the membrane, ribosomes/RNCs will only open those SecY channels that are in the correct orientation. Those in the opposite orientation will not bind ribosomes and are therefore silent in the electrophysiology experiments. YidC is also predominantly inserted into proteoliposomes in an inside-out orientation^49^. We also expect that a physiological interaction between SecY and YidC is only possible if both proteins are in their correct orientation. This is in line with our observation that only SecYEG-YidC channels are observable in a 1:1 stoichiometry. Only when an excess of SecYEG is reconstituted into the bilayer, we observe both smaller SecYEG-YidC and larger SecYEG channels.

Both our electrophysiological and crosslinking data are most easily explained by the assumption that one or more of the YidC TMs is already intercalated at the lateral gate of the empty SecY. There is up to now no structural information about a SecYEG-YidC complex, therefore it is currently unknown which part of YidC reaches into the channel. The first TM of YidC has not been crystallized so far because it is like the N-terminal part of the periplasmic domain disordered^50^. This probably indicates that the first TM is not in contact with the five TM-core of YidC, which would make it suitable for intercalating into the lateral gate. This would probably maintain YidC in an orientation in which its hydrophilic substrate binding groove would face the lateral gate and thus allow contact to substrates emerging from the Sec translocon. During co-translational protein insertion, the YidC residues serve to alter the hydrophobicity of the channel interior thereby modifying the release probability of TMs into the membrane. Since this kind of thermodynamic partitioning^51, 52^ is known to be susceptible to the exact location of amino acids in the pore and to groupings of hydrophobic or hydrophilic residues in it^53^, it must also be sensitive to the actual hydrophobicity of the wall lining residues. The release of some weakly hydrophobic TMs is thus promoted by rendering the pore less hydrophobic^19, 54, 55^. YidC’s hydrophilic groove with a conserved positively charged arginine residue in the membrane center^50^ would work nicely for that purpose. The involvement of this arginine residue in the RNC_FtsQ_-YidC-SecYEG pore may also explain the observed increase in anion selectivity as compared to the RNC_FtsQ_-SecYEG complex. This particular function of the arginine residue during SecYEG-mediated membrane insertion might also be the reason why deleting it had no effect on the YidC-only membrane insertion pathway in *E. coli* ^56^.

One puzzling observation of the current study is that the interaction of YidC with the channel interior is more pronounced in the absence of the accessory SecDF complex. SecDF probably functions as a pmf-powered chaperone system that facilitates late steps during protein translocation^22^. So far, SecDF has been mainly associated with SecA-dependent steps of protein transport^9, 26, 57^, but its contribution to membrane protein integration is largely unknown. In a stoichiometric SecYEG-YidC-SecDFYajC complex, SecDF were crosslinked to SecE, SecG and to the N-terminal half of SecY (helices 1–5)^25^. Thus, the SecDF binding site on SecYEG is very close or even partially overlaps with the binding site for YidC, which involves helices 2, 3, 7 and 8^20^. The contact of YidC with these helices is not significantly different in the absence or presence of SecDF^20^, but the observed crosslink probabilities suggest that YidC appears to have easier access to the channel interior when SecDF is depleted. This could reflect possible competition, in which SecDF disturbs YidC’s contact to the pore ring residues. However, competition is difficult to envision considering that SecDF are low abundant proteins with approx. 30 copies per *E. coli* cell compared to approx. 300–400 SecYEG copies and 3000 YidC copies^58^. A more likely explanation is provided by SecDF’s ability to facilitate transmembrane proton transport. SecDF could convert the proton gradient into an increment in transmembrane potential that in turn, may force conformational changes in SecYEG. This is in line with the enhanced co-translational insertion of the SecYEG- and YidC-dependent membrane protein CyoA in the presence of SecDF^25^ and the observation that SecDF influences SecYEG-YidC complex formation, although SecDF is not part of this complex^15^. Finally, this explanation is also supported by our previous observation that SecYEG-SecA complexes with a stalled translocation intermediate cease to conduct ions at high membrane potentials^40^. Assuming that the ion-conducting empty SecYEG-ribosome complex^39^ behaves similarly, this would explain YidC’s reduced access probability to the channel interior.

**Figure 7:**
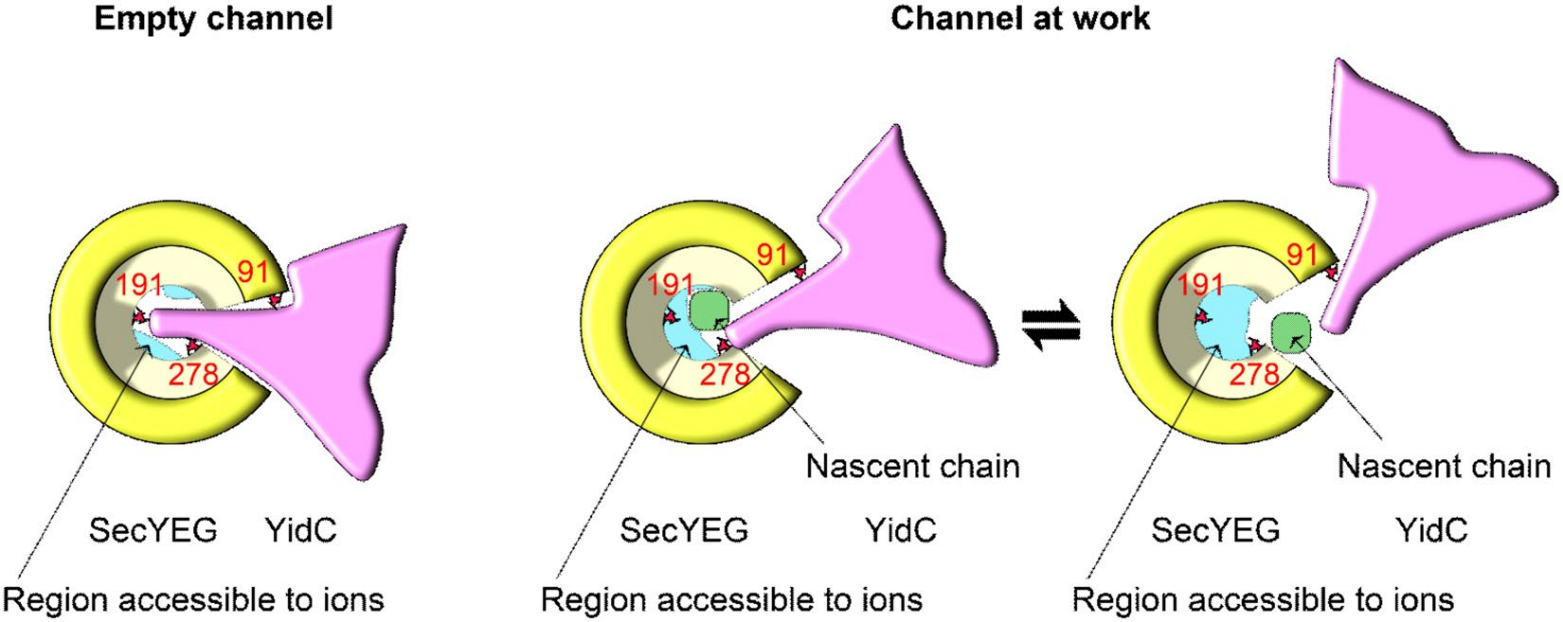
Model of the YidC-SecYEG interaction as visualized by crosslinks and electrophysiological experiments. *Left Panel*: The empty SecYEG-YidC complex allows YidC to crosslink to the three indicated positions (red) at SecYEG’s pore ring (yellow) or the lateral gate. Ribosome binding is required to elicit the formation of a transmembrane channel (cross-section in blue) that allows ion permeation at low membrane potentials. *Right Panel*: Upon insertion of a nascent chain (green), YidC is expelled to the outer rim of the SecYEG pore, thereby increasing the cross-section (ion conductivity) of the SecYEG channel. Crosslinks to the channel interior (I191) is sterically constrained in the presence of a nascent chain while crosslinks to the peripheral residues 91 and 278 are still observable. In order to release the nascent chain, the lateral gate must open, *i.e.* conceivably the contact between SecYEG and YidC is further weakened. This is in line with crosslink data showing conformational changes at the SecY(I91)-YidC interface^20^. In summary, YidC facilitates the partitioning of a nascent membrane protein into the lipid environment by reducing the hydrophobicity of the lateral gate.

We conclude that YidC facilitates membrane protein insertion by forming a heterotetrameric channel with SecYEG. The complex maintains the membrane barrier to ions at rest and opens up upon ribosome or RNC binding (Fig. 7). Although higher (physiological) membrane potentials are likely to close the channel for the passage of ions, the nascent chain may still be released through the lateral gate, which by virtue of its reduced hydrophobicity when YidC is intercalated, favors membrane partitioning of TMs that the SecYEG pore would otherwise preferentially release into the aqueous environment.

## Methods

### *In vivo* and *in vitro* pBpa cross-linking, protein purification

For *in vivo* cross-linking, *E. coli* BL21 and *E. coli* BL325 harboring the plasmids pSup-BpaRS-6TRN and pTrc99a-SecY(His)EG were grown at 30 °C in minimal medium in the presence of 1mM pBpa as previously described^20, 29^. After harvesting, the cells were washed once with 50 mM triethanolamine acetate (TeaOAc) pH 7.5 and incubated on ice for 30 minutes under a UV lamp in PBS buffer (137 mM NaCl, 2.7 mM KCl, 10 mM Na_2_HPO_4_, 1.76 mM NaH_2_HPO_4_, pH 7.6). Cells were lysed in a French pressure cell and membranes were prepared and solubilized with 1% DDM (n-Dodecyl β-D-maltoside) (Thermo Scientific, Dreieich, Germany). SecY was further purified via Talon® Affinity Resin (Clontech, Mountain View, CA) and its cross-linking partners were identified by immune detection. To perform the *in vivo* crosslinking in SecDF depleted cells, *E. coli* BL325 containing pSup-BpaRS-6TRN and pTrc99a-SecY(His)EG with the amber stop codon at the indicated positions within SecY were grown overnight on LB medium containing 0.2% arabinose. After a washing step, these cells were used to inoculate a 1l culture containing pBpa and either 0.2% arabinose (SecDF^+^) or 0.2% glucose (SecDF^−^). Cells were grown to an OD600 of 1.0 and SecY(His)EG expression was induced with 1 mM IPTG. Cells continued to grow up to an OD600 of 2.0 and were then harvested. UV exposure and purification of crosslinking products followed the above-described protocol.

For *in vitro* crosslinking, inner membrane vesicles (INVs) were prepared from *E. coli* cells expressing SecYpBpa in the presence of 1 mM pBpa according to the procedure described previously^20, 29^. For the crosslinking in the presence of RNCs, the ribosome-associated nascent chains were incubated with INV (4 μg/μl) on ice in INV buffer (100 mM TeaOAc pH 8, 250 mM Sucrose, 5 mM Mg(Ac)_2_, 0.1 mM PMSF (phenylmethylsulfonyl fluoride), 0.5 mM ZnCl_2_ and 0.1% Roche protease inhibitor cocktail) and UV irradiated for 20 min. The reactions were subsequently incubated with 0.2 M Na_2_CO_3_ at pH 11.6 on ice for 30 min. and the membranes were pelleted by centrifugation for 64 min. at 72,000 × g (TLA 55 rotor, Beckman). The membrane proteins were then solubilized with 1% DDM and SecY crosslinking products were purified via Talon Affinity resin and visualized by western blotting. The purification of ribosomes and INV via sucrose-gradient purification followed previously published protocols^36^.

### Purification of SecYEG and YidC for reconstitution into proteoliposomes

Sec_His_EYG and YidC were purified as reported previously with small changes^17, 39^. The SecY(R255E,R256E)EG mutant complex was generated by PCR using the Phusion PCR Kit (NE Biolabs, Frankfurt, Germany) with 5′-phosphorylated mutagenic oligonucleotides. (SecY(R255E,R256E)_for 5′-CAGCAAGGTGAGGAGGTCTATGCT-3′; SecY(R255E, R256E)_rev 5′-ACGTTTCGCGTAGTTTACCACAAT-3′) using pBAD-SecHisEYG as template. The SecY(R255E,R256E)EG complex was purified from TY0 cells as described below.

Protein purification was carried out using a Ni-NTA FF crude column (GE Healthcare) on an ÄKTA chromatography system. The equilibration/ wash buffer contained 50 mM Tris-HCl pH 7.5, 300 mM NaCl, 5 mM MgCl, 20 mM imidazole, 0.03% DDM (Affymetrix Antrace), 10% glycerol and His-tagged proteins were eluted with a linear gradient from 20 to 500 mM imidazole. Dried *E. coli* polar lipid extract (Avanti Polar Lipids) was rehydrated in buffer A (50 mM TeaOAc pH. 7.5, and 50 mM DTT) to a final concentration of 100 mg/ml and sonicated. Proteoliposomes were prepared by mixing lipids (final concentrations of 0.1 mg/ml) and 0.85% (w/v) n-octyl-β-D-glycoside with 1.5 μM purified and DDM-solubilized proteins. The reconstitution mix was incubated for 20 min at 4 °C. Samples were subsequently dialysed (spectrapor membrane tubing, 6–8 kDa) against 50 mM TeaOAc, pH 7.5 and, 1 mM DTT. The proteoliposomes were pelleted (1 h, 210 000 × g) and re-suspended in 50 mM TeaOAc, pH 7.5 and, 1 mM DTT to a final protein concentration of 5 μM. For proteoliposomes containing both SecYEG and YidC, SecYEG and YidC were reconstituted in a 1:1 molar ratio.

The SecYEG complex, carrying a single cysteine residue at position 204, was purified as described^39^. The expression of the SecYEG complex in *E. coli* C43 (DE3) cells was induced with arabinose for 4 h at 37 °C. The membranes were solubilized in 1% DDM and the extract was then passed over a Ni^2+^-chelating column, concentrated and incubated with TCEP (Tris(2-carboxyethyl)phosphine, Fluka) for 5 min at 4 °C. Atto488-maleimide (100 μM) was added and kept under steady mixing at 4 °C for 2 hours. The sample was diluted with solubilization buffer (300 mM NaCl, 0.6 mM DDM, 10% Glycerol, 20 mM Tris, pH 7.5) to reduce the imidazole concentration to <10 mM and mixed with Ni-NTA beads for 30 min at 4 °C. After washing with 50 ml wash buffer (300 mM NaCl, 20 mM imidazole, 0.6 mM DDM, 10% Glycerol, 20 mM Tris, pH 7.5), the protein was subjected to size-exclusion chromatography. Purified SecYEG complexes were stored at –80 °C in TNG buffer (10 mM Tris-Cl, pH 8.0, 150 mM NaCl, 10% glycerol, 10 mM DTT, 0.03% DDM). Protein concentrations were determined by fluorescence correlation spectroscopy. The purified SecYEG complex was reconstituted into proteoliposomes using Bio-beads SM2 (Bio Rad) for detergent removal. In brief, the reconstitution mixture was prepared at room temperature by sequentially adding 20 mg/ml of *Escherichia coli* polar phospholipids (Avanti Polar Lipids, Alabaster, AL) in 50 mM K-HEPES pH 7.5, 6% deoxy Big-CHAP (Affymetrix Anatrace, Cleveland, OH) and purified protein in detergent (Protein to Lipid ratio of 1:36 to 1:100). The mixture was 8-fold diluted in 50 mM HEPES pH 7.5, 200 mM K-acetate, 12.5% glycerol. Biobeads were added to the mixture and incubated overnight on a shaker. The proteoliposomes were harvested by ultracentrifugation (80 min at 100,000 × g) and resuspended in an assay buffer at a concentration of 5–10 mg/ml. The assay buffer contained 50 mM HEPES pH 7.0, 10% glycerol and cOmplete protease inhibitor cocktail (Roche).

### Ribosome expression and purification

Tetra-(His)_6_-tagged ribosomes from *E. coli* JE28 strain were purified as described previously^59^. An overnight culture of *E. coli* JE28 was used to inoculate 1 liter LB-medium supplemented with 50 μg/ml kanamycin. The cells were grown to an OD600 of 1.0 at 37 °C. Thereafter the culture was kept at room temperature for 1 h before shifting it to 4 °C for another hour to produce run-off ribosomes. The cells were harvested by centrifugation at 4,000 rpm for 30 min. For purification the cell pellet was re-suspended in lysis buffer (20 mM Tris-HCl pH 7.6, 10 mM MgCl_2_, 150 mM KCl, 30 mM NH_4_Cl) with 0.5 mg/ml lysozyme, 10 μg/ml DNAse I and 20 μM puromycin and lysed using a BeadBeater (BioSpec). The lysate was then clarified by centrifugation and passed over a Ni^2+^-chelating column. The ribosomes were eluted with 150 mM imidazole and then dialyzed overnight against lysis buffer. The ribosomes were then pelletized by ultracentrifugation and re-suspended in 500 mM NH_4_Cl, 50 mM Tris-acetate and 25 mM Mg-acetate giving a final concentration of 10–20 mg/ml. pH was adjusted to 7.2. All buffers were supplemented with cOmplete protease inhibitor cocktail (Roche).

### Purification of FtsQ-RNCs

Elongation-arrested 176 amino acid-long nascent chains consisted of the 101 N-terminal amino acids of the membrane protein FtsQ^60^. Between amino acids 27 and 28 of FtsQ, a calmodulin binding peptide was inserted that was flanked on both sides by Gly - Gly spacers. A TEV cleavage site and the stalling sequence SecM represented the C-terminus of the nascent chain. We used Bad22 as the vector backbone for expression in his-tagged - ribosome producing JE28 *E. coli* cells^59^. The JE28 cells were grown to an OD of 1.0 in 2xYT medium with kanamycin (30 mg/l) and ampicillin (100 mg/l). RNC expression was induced by arabinose (2 g/l). The JE 28 cells expressing cells were lysed in Ribo Basic (20 mM Tris pH 7.6, 10 mM MgCl_2_, 150 mM KCl, 30 mM NH_4_Cl) with a French press followed by centrifugation at 25000 g. In a first purification step, we incubated the supernatant with calmodulin agarose in the presence of 2 mM CaCl_2_ for 1 h. After washing copiously with Ribo Basic (+2 mM CaCl_2_) the RNCs were eluted with 20 mM Tris pH 7.6, 150 mM KCl, 30 mM NH_4_Cl and 2 mM EGTA. To get rid of peptide chains that were not bound to ribosomes, a second purification step followed: The eluate of the first purification step was bound to NiNTA –Agarose (Quiagen), washed with 5 mM and eluted in 150 mM imidazole containing Ribo Basic Buffer.

For *in vitro* crosslinking, N-terminally His-tagged RNCs carrying the first 102 amino acids of FtsQ followed by an HA tag and a TnaC stalling sequence (pftsQ-tnaC) were expressed in *E. coli* KC6(DE3) (kindly provided by R. Beckmann, Ludwig Maximilian University of Munich, Germany). The RNC were generated by growing cells in LB-medium (lysogeny broth) to an OD of 0.5 and induced with 1 mM IPTG for 1 h. The cells were harvested and resuspended in buffer A (50 mM Hepes pH 7.5, 250 mM KOAc pH 7.5, 25 mM Mg(OAc)_2_ pH 7.5, 250 mM sucrose and 0.1% DDM) containing additionally 1 mM tryptophan and 250 μg/ml chloramphenicol. After cell breakage using a French Press, the cell debris was removed by centrifugation for 20 min. at 16,000 rpm in an SS34 rotor. The lysate was further purified by centrifugation for 17 hours on sucrose cushion (buffer A containing 750 mM sucrose) at 22.000 rpm (Beckmann Ti 50.2 rotor). The pellet containing the FtsQ-RNC was resuspended in buffer A and incubated for one hour with Talon Metal Affinity Resin (BD) (0.5 ml slurry/ 1 L of culture). After 4 subsequent washing steps, the RNCs were eluted with in buffer A supplemented with 100 mM imidazole. Finally, the ribosome-associated nascent chains were pelleted for 2 h at 86,000 g and resuspended in INV buffer (100 mM TeaOAc pH 8, 250 mM sucrose, 5 mM Mg(Ac)_2_).

### Reconstitution of SecYEG and YidC/SecYEG into planar bilayers

“Solvent-free” planar lipid bilayers were folded by raising the level of two adjacent aqueous solutions over the dividing 100–200 μm-wide aperture in a Teflon septum with *E. coli* polar lipid extract (Avanti Polar Lipids, Alabaster, AL) monolayers on top^61^. Fusion of proteoliposomes containing either reconstituted SecYEG or YidC/SecYEG to the free standing planar lipid membranes (Fig. 4) was facilitated by raising the osmotic pressure in one of the compartments (*cis*) of the Teflon chamber^39, 40^. The *cis* compartment also contained the proteoliposomes and the ribosomes/RNCs. SecYEG and YidC were present in proteoliposomes in a 1:1 molar ratio (2.5 nM each). The ribosome concentration was 150 nM and the FtsQ-RNC concentration 5 nM. The second compartment (*trans*) contained only the buffer. It consisted of 50 mM K-HEPES, pH 7.5 and 150 mM KCl. Protein reconstitution into planar bilayers in symmetrical salt conditions (Fig. 3) was performed as described before^44^. In brief, a mixture of proteoliposomes and empty lipid vesicles was added to the *cis* compartment. The *trans* compartment solely contained empty lipid vesicles in buffer. After allowing the formation of monolayers on top of the suspension, planar bilayers were folded as described above.

### Single ion channel measurements

Ag/AgCl reference electrodes were immersed into the buffer solutions on both sides of the planar bilayers. The command electrode of the patch clamp amplifier (model EPC9, HEKA electronics, Germany) was immersed into the cis compartment and the ground electrode into the *trans* compartment. The recording filter for the transmembrane current was a 4 pole Bessel with −3dB corner frequency of 0.1 kHz. The raw data we attained were analyzed using the TAC software package (Bruxton Corporation, Seattle, WA). Gaussian filters of 12 Hz were applied to reduce noise.

### Calculation of channel ion selectivity

The anion (Cl^−^) to cation (K^+^) permeability ratio *r* (P_Cl_/P_K_) is calculated from *ψ*_r_ (Fig. 3) using Goldman’s equation for bi-ionic potentials:

1 $${\psi }_{r}=\frac{RT}{F}\,\mathrm{ln}(\frac{{K}_{cis}^{+}+\frac{{P}_{Cl}}{{P}_{K}}C{l}_{trans}^{-}}{{K}_{trans}^{+}+\frac{{P}_{Cl}}{{P}_{K}}C{l}_{cis}^{-}})$$

where ${K}_{cis}^{+}$, ${K}_{trans}^{+}$, $C{l}_{cis}^{-}$, and $C{l}_{trans}^{-}$ indicate the ion concentrations on the two sides (termed *cis* and *trans*) of the membrane. We account for the osmotic water flow within the unstirred near-membrane layers which concentrates the solution on one hypoosmotical side and dilutes it on the hyperosmotical side of the membrane. For membranes with conductivities in the range of tens of pA, this effect does not usually exceed 10%^62^. Therefore we assumed the bulk KCl gradient of 450 to 150 mM to correspond to a 230 mM gradient across the lipid bilayer proper.

### Estimating pore diameter

We used channel conductance to obtain a rough estimate of pore diameter as previously described^44^:

2 $$\frac{1}{g}=(l+\frac{\pi d}{4})\frac{4}{\sigma \pi {d}^{2}}$$

where *d, l* = 3 nm, and σ = 3.7 S/m correspond to channel diameter, channel length and conductivity of the solution in the pore, respectively.

## Electronic supplementary material

Supplementary Text and Figures
