# Supplementary material for: YidC and SecYEG form a heterotetrameric protein translocation channel
Source: Sci Rep. 2017 Mar 7;7:101. doi: 10.1038/s41598-017-00109-8 (PMC5427846; doi:10.1038/s41598-017-00109-8)
Supplement: Supplementary file 1 — Supplementary Text and Figures [file 41598_2017_109_MOESM1_ESM.pdf]

Supplementary material to

YidC and SecYEG form a heterotetrameric protein translocation channel

Ilie Sachelaru[1,2,+], Lukas Winter[3,+], Denis Knyazev[3,+], Mirjam Zimmermann[3], Andreas Vogt[1,2,4], Roland Kuttner[3], Nicole Ollinger[3], Christine Siligan[3], Peter Pohl[3,*] and Hans-Georg Koch[1,4*]

[1]Institut für Biochemie und Molekularbiologie, ZBMZ, Faculty of Medicine and [2]Fakultät für Biologie, Albert-Ludwigs-Universität Freiburg, Stefan Meier Str. 17; 79104 Freiburg, Germany

[3]Institute of Biophysics, Johannes Kepler University Linz, Gruberstrasse 40, A-4020 Linz, Austria;

[4]Spemann-Graduate School of Biology and Medicine (SGBM), Albert-Ludwigs-Universität Freiburg, 79104 Freiburg, Germany

Supplementary Results

The SecYEG-YidC interaction appears to be redox-sensitive

In the crosslink experiments, we noticed that in all UV-exposed samples slightly more YidC co-purified with SecY, compared to the non-exposed sample. This was surprising as all samples contained comparable SecY amounts as verified by western blotting (see Figs. 1 & 2). This was further analyzed by performing the crosslinking after pre-treatment of the samples. As UV-exposure has been shown to also induce protein-RNA crosslinks, we analyzed whether increased co-purification was still detectable after RNAse A treatment. However, this treatment did not change the amount of YidC that co-purified with SecY after UV-exposure, indicating that increased co-purification is not the result of an RNA-stabilized SecYEG-YidC complex (Supplementary Figure 1). However, when the samples were pretreated with 50 mM DTT, the amount of YidC co-purifying with SecY was significantly reduced and comparable to the YidC amount in the UV-free sample. This indicates that the SecY-YidC interaction is redox-sensitive and that UV-exposure stabilizes this redox-sensitive interaction. However, this was not further analyzed in the current study.

SecYEG channel opening depends on a specific SecY-ribosome interaction.

For validating that channel opening by non-translating ribosomes depends on a specific SecY-ribosome interaction, we made use of the SecY(R255E, R256E)EG mutant complex. In this SecY mutant, two positively charged arginine residues within the cytosolic loop C4 are replaced by negatively charged glutamate residues. This interferes with the SecY-ribosome interaction both in detergent solution[1] and in inner membrane vesicles[2]. The SecY(R225E,R256E)EG complex was purified and reconstituted into proteoliposomes. Fluorescence correlation spectroscopy of Atto488-labelled SecYEG-proteoliposomes and

SecY(R255E,R256E)EG proteoliposomes revealed similar reconstitution efficiencies for wild type and mutant SecYEG complexes (4.1 and 4.6 complexes on average per vesicle, respectively) (Supplementary Figure 2).

A transmembrane salt gradient was then used to fuse these proteoliposomes with a planar lipid bilayer for electrophysiology experiments in the presence of ribosomes, as described in the main body of the manuscript. Ribosomes were purified in the presence of puromycin, which releases all nascent proteins from ribosomes and thus ensures a homogenous population of non-translating ribosomes. In contrast to the wild type SecYEG complex, we observed no channel activity of the SecY(R255E,R256E)EG complex, when incubated with similar ribosome concentrations (Supplementary Figure 3). These data validate that channel opening requires a specific ribosome-SecY interaction, which involves previously identified SecY residues important for ribosome binding. Furthermore, the data demonstrate that channel activity is not inflicted by the ribosome concentration or any contaminants therein.

Supplementary Figure Legends

Supplementary Figure 1: **Co-purification of YidC with SecYEG is reduced by DTT**. *In vitro* crosslinking was performed as described in the legend to Figure 1, with the exception that INV were either pretreated with 1mg/ml RNAseA or with 50 mM DTT for 15 min at 30°C. After this pretreatment, samples were UV-exposed and subsequently extracted with sodium carbonate (0.4 mM, pH 9.6) to remove DTT and RNAseA. After centrifugation the pellet fraction was used for SecYEG purification as described in Material and Methods.

Supplementary Figure 2. **The wild type SecYEG channel and the mutant SecYEG channel reconstituted with equal efficiency into lipid vesicles.** Shown are autocorrelation functions $G(\tau)$ that were obtained by fluorescence correlation spectroscopy. Both the wild type and mutant channels were labeled with ATTO488 in position A204C. First a suspension of proteoliposomes was measured (black dots). A conventional model for a one component three-dimensional diffusion $G(\tau) = 1 + \frac{1}{\mathrm{N}(1+\tau/\tau_D)}$ was fitted to the raw data (blue line). N and τ_D denote the number of particles N=1/(G(0)-1) and the residence time of the particles in the confocal volume. Subsequently the proteoliposomes were diluted by detergent (2 mass % OG, 3 % deoxyBigCHAP) and the resulting micellar solution was again subjected to fluorescence correlation spectroscopy (grey dots). The same model was fitted to the raw data (red line). Panel **A** shows the experiment with the wild type channel, panel **B** shows the experiment with the mutant channel. The increase in particle number that was observed after vesicle solubilization corresponds to 4.1 channels and 4.6 channels per vesicle in panels A and B, respectively. The calculations assume that every Atto488 labeled micelle holds exactly one channel. The buffer contained 150 mM KCl and 50 mM K-HEPES (pH 7.5) in both cases.

Supplementary Figure 3. **A SecYEG mutant deficient in ribosome binding does not exhibit channel activity when exposed to ribosomes.** The transmembrane potential was equal to - 35 mV (the ribosome containing compartment was negative - mimicking the *in vivo* situation). During the last steps of the ribosome purification process, 20 µM puromycin was added to ensure that no nascent peptide chains were bound. **A.** Fusion of vesicles with ribosome-activated wild type SecYEG channels to the planar bilayer resulted in a stepwise conductivity increase. **B**. The corresponding current histogram allows discerning the resulting distinct conductivity levels of the planar bilayer. The length of the scale bar on the right (x-axis) represents the number of counts of a given current level (y-axis) from A. **C**. Repeating the procedure outlined in **A** with the mutant channel SecY(R255E, R256E)EG that is deficient in ribosome binding did not result in channel activity and ribosomes were unable to activate the channel. **D**. The corresponding current histogram does not show any conductivity levels beside the base line. For all other conditions please see Figure 4 of the main manuscript. Scale bars for C & D are the same as for A & B.

Supplementary References

1 Menetret, J. F. *et al.* Ribosome binding of a single copy of the SecY complex: implications for protein translocation. *Molecular cell* **28**, 1083-1092, doi:10.1016/j.molcel.2007.10.034 (2007).

2 Wu, Z. C., de Keyzer, J., Kedrov, A. & Driessen, A. J. Competitive binding of the SecA ATPase and ribosomes to the SecYEG translocon. *The Journal of biological chemistry* **287**, 7885-7895, doi:10.1074/jbc.M111.297911 (2012).

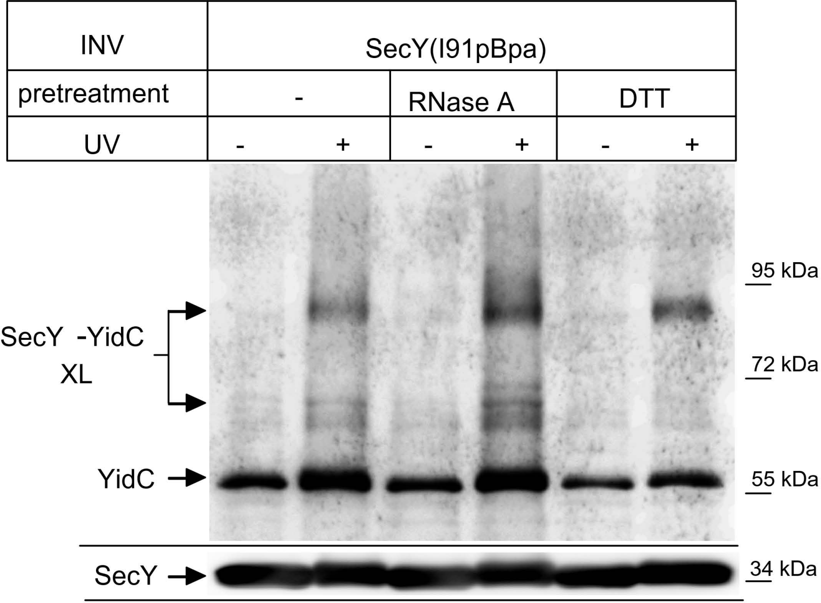

(Sachelaru et al., Fig. S 1)

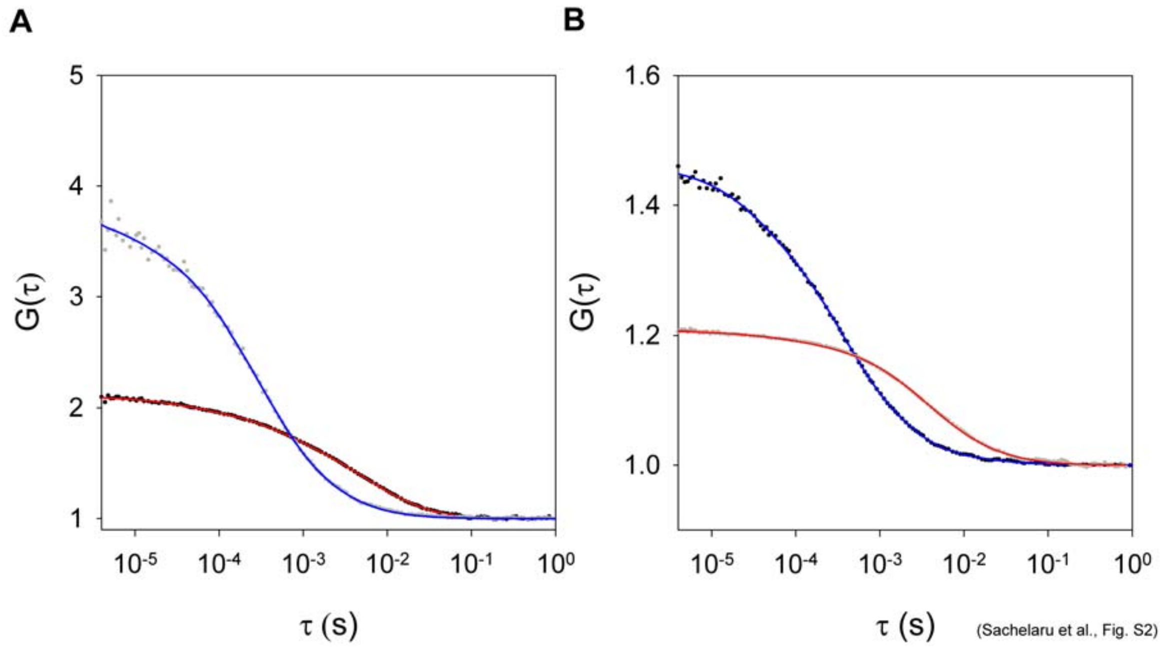

(Sachelaru et al., Fig. S2)

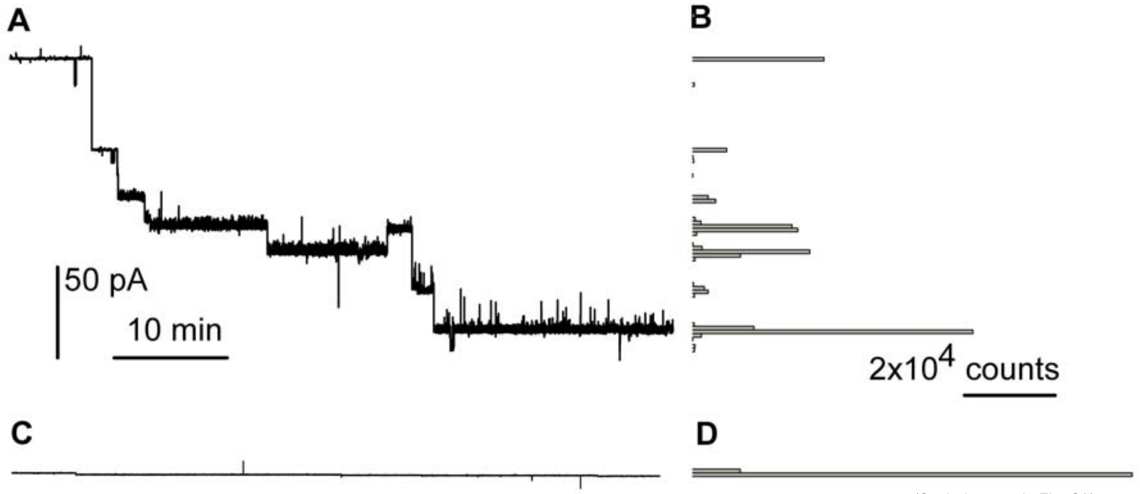

(Sachelaru et al., Fig. S3)
